# Rituximab therapy for intractable pemphigus: A multicenter, open‐label, single‐arm, prospective study of 20 Japanese patients

**DOI:** 10.1111/1346-8138.16597

**Published:** 2022-10-04

**Authors:** Jun Yamagami, Yuichi Kurihara, Takeru Funakoshi, Yasuko Saito, Ryo Tanaka, Hayato Takahashi, Hideyuki Ujiie, Hiroaki Iwata, Yoji Hirai, Keiji Iwatsuki, Norito Ishii, Jun Sakurai, Takayuki Abe, Ryo Takemura, Naomi Mashino, Masahiro Abe, Masayuki Amagai

**Affiliations:** ^1^ Department of Dermatology Keio University School of Medicine Tokyo Japan; ^2^ Department of Dermatology Tokyo Women's Medical University Tokyo Japan; ^3^ Department of Dermatology, Faculty of Medicine and Graduate School of Medicine Hokkaido University Sapporo Japan; ^4^ Department of Dermatology Gifu University Graduate School of Medicine Gifu Japan; ^5^ Department of Dermatology Okayama University School of Medicine Okayama Japan; ^6^ Department of Dermatology Kurume University School of Medicine Kurume Japan; ^7^ Center for Innovative Clinical Medicine Okayama University Hospital Okayama Japan; ^8^ Biostatistics, Clinical and Translational Research Center Keio University School of Medicine Tokyo Japan; ^9^ Yokohama City University School of Data Science Yokohama Japan; ^10^ Prescription Products Development Department Zenyaku Kogyo Co., Ltd. Tokyo Japan

**Keywords:** anti‐desmoglein antibody, corticosteroid tapering, refractory pemphigus, rituximab

## Abstract

This was a multicenter clinical trial of rituximab, a chimeric monoclonal IgG antibody directed against CD20, for the treatment of refractory pemphigus vulgaris and pemphigus foliaceus. In total, 20 patients were treated with two doses of rituximab (1000 mg; 2 weeks apart) on days 0 and 14. The primary end point was the proportion of patients who achieved complete or partial remission on day 168 following the first rituximab dose. Of the 20 enrolled patients, 11 (55%) and four (20%) achieved complete and partial remission, respectively; therefore, remission was achieved in a total of 15 patients (75.0% [95% confidence interval, 50.9%–91.3%]). It was demonstrated that the remission rate was greater than the prespecified threshold (5%). In addition, a significant improvement in clinical score (Pemphigus Disease Area Index) and decrease in serum anti‐desmoglein antibody level were observed over time. Four serious adverse events (heart failure, pneumonia, radial fracture, and osteonecrosis) were recorded in two patients, of which only pneumonia was considered causally related with rituximab. The level of peripheral blood CD19‐positive B lymphocytes was decreased on day 28 after rituximab treatment and remained low throughout the study period until day 168. Our results confirm the efficacy and safety of rituximab therapy for refractory pemphigus in Japanese patients.

## 1 INTRODUCTION

Pemphigus is an autoimmune bullous disease characterized by skin and mucous membrane blisters and erosions with intraepidermal blister formation caused by IgG autoantibodies directed against adhesion proteins, desmoglein 1 (Dsg1) and desmoglein 3 (Dsg3), located in the desmosomes between epidermal keratinocytes.^1^, ^2^ For patients with moderate to severe pemphigus, guidelines recommend high‐dose systemic corticosteroids, such as prednisone (1 mg/kg per day) as the first‐line treatment.^3^, ^4^, ^5^ The goal of treatment is remission, defined as symptom freedom on ≤10 mg/day of prednisone or equivalent dose and minimal adjuvant therapy (e.g., immunosuppressive agents).^6^ Japanese guidelines recommend that pemphigus treatment be divided into two phases: consolidation and maintenance.^3^ During the consolidation phase (initial therapy), treatment is adjusted until the disease is controlled, including tapering of the corticosteroid dose. During the maintenance phase (maintenance therapy), treatment is maintained and the corticosteroid dose is tapered. Additional therapies, such as plasma exchange and high‐dose intravenous immunoglobulin, should be administered if initial therapy is not sufficient in controlling disease activity. A recent study reported that >90% of patients with pemphigus achieved remission within the first 2 years of guideline‐based treatment, suggesting the effectiveness of such a treatment plan.^7^ However, there are still unmet needs in pemphigus treatment, including the inability to achieve remission in ≈10% of patients and the high incidence (≈80%) of treatment‐related side effects, such as diabetes, osteoporosis, and serious infections, which may be fatal.^8^, ^9^ Therefore, targeted therapies against autoantibody production are required to improve the treatment of pemphigus.

Rituximab is a chimeric human‐mouse IgG monoclonal antibody that binds to the transmembrane antigen CD20 expressed from the pre–B‐cell stage to the preplasma cell stage.^10^ The binding of rituximab to CD20 leads to B‐cell depletion through various mechanisms, including complement‐dependent cytotoxicity, antibody‐dependent cell‐mediated cytotoxicity, and apoptosis.^11^ Rituximab is approved for the treatment of B‐cell lymphoma and autoimmune diseases, including rheumatoid arthritis and anti‐neutrophil cytoplasmic antibody–associated vasculitis, although the indications vary between regions. Since 2001, several case reports and case series have reported remarkable therapeutic effects of rituximab in pemphigus patients.^12^, ^13^, ^14^, ^15^ Rituximab has already been administered to >500 patients and is recommended as the standard treatment for refractory cases of pemphigus by the European guidelines.^16^, ^17^ A prospective, multicenter, parallel‐group, open‐label randomized trial showed significant therapeutic effect of rituximab for pemphigus compared with placebo, which prompted the approval of rituximab for pemphigus treatment by health insurance in the United States and Europe in 2018 and 2019, respectively.^18^

In Japan, we conducted an exploratory study of the efficacy and safety of rituximab in patients with autoimmune bullous diseases refractory to corticosteroid therapy since 2009 and found that rituximab reduced the disease activity and decreased the corticosteroid dose requirement.^19^ Based on previous reports, we performed an investigator‐initiated clinical trial to validate the efficacy and safety of rituximab for the treatment of refractory pemphigus in patients who did not achieve remission with other treatments.

## 2 METHODS

### 2.1 Study design

**FIGURE 1:**
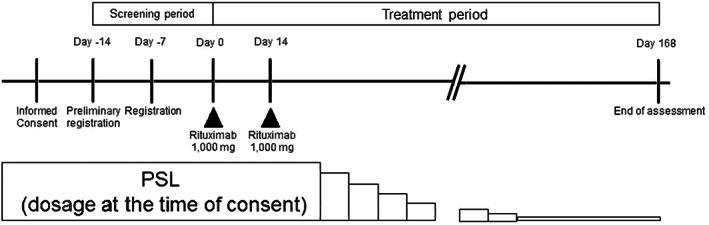
Study protocol.

We performed a single‐arm, prospective, open‐label, interventional trial at Keio University Hospital, Hokkaido University Hospital, Okayama University Hospital, and Kurume University Hospital, Japan. The study was conducted in accordance with the Declaration of Helsinki and the International Council for Harmonization: Good Clinical Practice and was approved by the institutional review board of each institution. Written informed consent was obtained from the patients. This study was registered at University Hospital Medical Information Network (UMIN) (UMIN000024265). The study consisted of screening (2 weeks; days −14 to day −1), treatment (2 weeks; days 0–14), and follow‐up (22 weeks; days 15–168), as shown in Figure 1.

### 2.2 Patients

Patients diagnosed with pemphigus vulgaris (PV) or pemphigus foliaceus (PF) in accordance with the Japanese guidelines for the management of pemphigus and disease relapse during corticosteroid taper were included in the study.^3^ Patients with a Pemphigus Disease Area Index (PDAI) score of 1 to 50 at the time of obtaining consent, daily corticosteroid dose of 15 to 30 mg/day (prednisone equivalent), no change in dose during 14 days of the screening period before study registration, and unchanged or worsened PDAI score during the screening period were included.^20^ We excluded patients receiving consolidation therapy, such as increasing doses of immunosuppressive agents, intravenous immunoglobulin, steroid pulse therapy, or plasma exchange in the prior 8 weeks. For safety reasons, we also excluded patients who were pregnant, who had undergone surgery within the prior 4 weeks, who had taken antibiotics within the prior 8 weeks, and/or those with a history of allergic reactions against chimeric human‐mouse IgG monoclonal antibody, severe organ disorders (such as chronic obstructive pulmonary disease, asthma, heart disease, and hypertension), active or chronic infection, deep‐seated infection (such as abscess, fasciitis, and osteomyelitis) within the past 1 year, or malignant tumors.

### 2.3 Treatment

Patients received intravenous rituximab at a dose of 1000 mg on days 0 and 14, and continued the prednisone adjuvant and immunosuppressive agents received at the time of enrollment. The patients received premedication with antihistamines and antipyretic analgesics 30 minutes before rituximab infusions to prevent infusion reactions. Starting 2 weeks after the second rituximab dose (day 48), prednisone doses were reduced following the predefined schedule and reached a dose of 10 mg/day by day 112 (Figure 1). Adjuvant immunosuppressive agents were continued without dose modification, if tolerated. Patients were evaluated weekly during rituximab treatment (days 0–14) and every 2 to 4 weeks thereafter during the observation period (days 15–168).

### 2.4 End Points

The primary end point was the rate of complete remission (CR) + partial remission (PR) at day 168 (i.e., 24 weeks after the first rituximab infusion). CR was defined as the absence of new or established lesions for at least 8 weeks while the patient was receiving minimal therapy (prednisone ≤ 10 mg/day alone or with minimal adjuvant immunosuppressive therapy), whereas PR was defined as only transient lesions that resolved within 1 week, with no treatment or only topical corticosteroids without increasing the systemic prednisone dose.^6^

The secondary end points were PDAI change, autoantibody titers against Dsg1 and Dsg3, and peripheral blood B‐ and T‐cell counts. Safety events were recorded using MedDRA/J version 22.0, and their severity was graded according to the common terminology criteria for adverse events version 4.0. Other end points included pharmacokinetic analysis, expression rate of antidrug antibody (ADA; treatment‐induced anti‐rituximab antibody), and immunoglobulin levels.

### 2.5 Statistical analysis

Based on previous studies, we expected a 50% remission rate at day 168 following rituximab administration and a spontaneous remission rate of 5% (threshold).^19^ Under these assumptions, eight patients were needed to have ≥80% power to demonstrate that the true remission rate is greater than the threshold. In the protocol, the target number of patients for enrollment was 10, and it was stated that the enrollment would be continued after achieving the target until the end of the accrual period. All enrolled patients were included in the primary efficacy analysis population (full analysis set). The 95% confident intervals (CIs) for proportions were calculated using the Clopper‐Pearson method. If the 95% lower confidence limit was greater than the prespecified threshold (5%), the null hypothesis (H0: remission rate = 5%) was rejected. Patients who withdraw from the study before day 168 (i.e., week 24) were considered as nonresponders. The significance level for tests was set at two‐tailed 5%. All statistical analyses were performed using SAS version 9.4 (SAS Institute Inc). This study was registered with the UMIN Center Trials registry, number UMIN000024265.

## 3 RESULTS

### 3.1 Patient characteristics

Between 2016 and 2018, a total of 20 patients (11 with PV, eight with PF, and one with PV/PF overlap) were enrolled in the study. The clinical characteristics of the patients are presented in Table 1. The median age was 48 years (24–72 years). The median PDAI was 10.5 (2–27). The median titers of autoantibodies against Dsg1 and Dsg3 were 276.0 U/ml (27.7–1300 U/ml) and 499.0 U/ml (40.1–973.0 U/ml), respectively. At baseline, the median prednisone dose was 17.8 mg/day (15–30 mg/day), and 17 of 20 patients were receiving adjuvant immunosuppressive agents (mycophenolate mofetil, *n* = 1; azathioprine, *n* = 16, cyclosporin, *n* = 3) in addition to prednisone.

**TABLE 1:** Characteristics of patients in the present study

	Patients *n* = 20
Age, y	
Mean ± SD	48.9 ± 10.9
Median (minimum–maximum)	48.0 (24–72)
Sex	
Male	14 (70.0%)
Female	6 (30.0%)
Type of pemphigus	
PV	11 (55.0%)
PF	8 (40.0%)
PV/PF overlap	1 (5.0%)
Severity of disease (PDAI score)	
Mean ± SD	11.7 ± 7.7
Median (minimum–maximum)	10.5 (2–27)
Serum anti‐Dsg antibody	
Dsg1 (*n* = 18)	
Mean ± SD	343.34 ± 327.16
Median (minimum–maximum)	276.00 (27.7–1300.0)
Dsg3 (*n* = 10)	
Mean ± SD	513.54 ± 366.51
Median (minimum–maximum)	499.00 (40.1–973.0)
Concomitant therapy	
Yes	17 (85.0%)
No	3 (15.0%)

Abbreviations: Dsg1, desmoglein 1; Dsg3, desmoglein 3; PDAI, pemphigus disease area index; PF, pemphigus foliaceus; PV, pemphigus vulgaris; SD, standard deviation.

### 3.2 Response to rituximab

**FIGURE 2:**
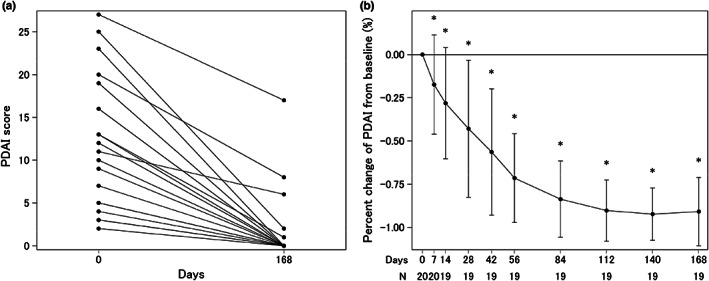
Changes in Pemphigus Disease Area Index (PDAI) over the course of the current study. (a) Changes in PDAI score in all 20 patients enrolled. There were two cases each with PDAI values of 3 at baseline and 0 at day 168, and two cases with values of 5 at baseline and 0 at day 168. The data for day 168 of one of the patients who was unable to continue because of pneumonia are not shown. For the above reasons, 17 lines are visible on this figure. (b) Changes in ratio of PDAI from baseline. Data are mean (standard deviation). *p < 0.05 relative to baseline by Student *t* test.

**FIGURE 3:**
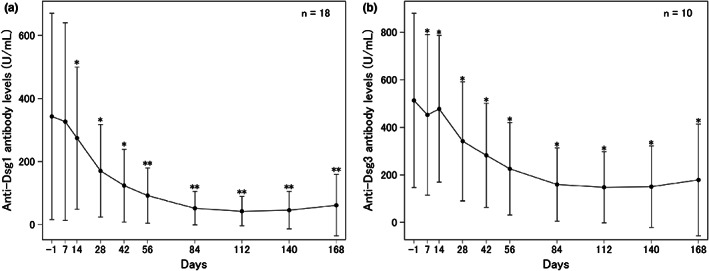
Changes in serum anti‐desmoglein (Dsg) autoantibodies over the course of the current study. (a) Anti‐Dsg1 antibody levels. (b) Anti‐Dsg3 antibody levels. Data are mean (standard deviation). *p < 0.05, **p < 0.01 relative to baseline by Student *t* test.

After 168 days of the first rituximab infusion (primary end point), 15 of 20 patients (75.0% [95% CI, 50.9%–91.3%]) achieved remission, of whom 11 (55.0%) and four (20.0%) patients achieved CR and PR, respectively. It was demonstrated that the remission rate was greater than the prespecified threshold (5%). The PDAI score decreased significantly from 11.7 ± 7.7 (mean ± standard deviation [SD]) at baseline to 1.8 ± 4.3 on day 168 (*p* < 0.001, Figure 2). The PDAI score improved significantly at all study points after day 7 and remained at the lowest level (<2) between day 112 and 168 (Figure 2b). In addition, serum anti‐Dsg autoantibodies decreased over time. The anti‐Dsg1 autoantibody titer decreased significantly from 309.16 ± 326.86 U/ml at baseline to 259.94 ± 228.03 U/ml, 161.75 ± 147.68 U/ml, 87.21 ± 87.64 U/ml, and 49.24 ± 52.91 U/ml on days 14, 28, 56, and 84, respectively. The anti‐Dsg3 autoantibody titer decreased significantly from 257.52 ± 364.18 U/ml at baseline to 227.29 ± 319.47, 162.45 ± 241.28, 107.89 ± 173.43, and 76.54 ± 131.02 U/ml on days 14, 28, 56, and 84, respectively. The serum anti‐Dsg1 and anti‐Dsg3 autoantibody titers remained significantly low after day 14 compared with baseline (Figure 3). All patients achieved successful corticosteroid tapering from a median of 17.8 mg/day (15–30 mg/day) at baseline to 10 mg/day at day 112 (i.e., week 16), and maintained this level throughout the study until day 168 (i.e., week 24).

**FIGURE 4:**
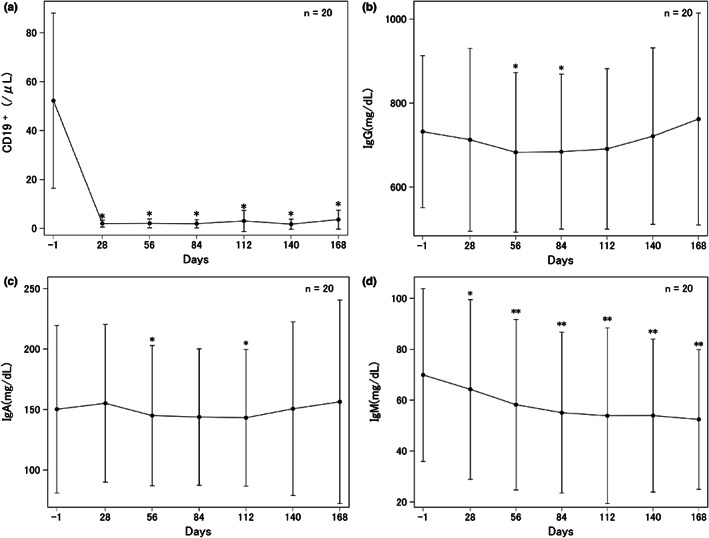
Changes in B‐cell counts and immunoglobulin levels over the course of the current study. (a) Changes in CD19‐positive cells in the peripheral blood. (b) Changes in IgG. (c) Changes in IgA. (d) Changes in IgM. Data are mean (standard deviation). *p < 0.05, **p < 0.001 relative to baseline by Student *t* test.

Peripheral blood B‐cell counts, as measured based on CD19‐positive cells, showed a significant decrease on day 28 (1.9 ± 1.4/μl) compared with baseline (52.3 ± 35.8/μl, *p* < 0.001); the counts remained low throughout the study until day 168 (Figure 4a). The number of CD3‐positive T cells did not change (data not shown). Serum immunoglobulin levels (IgG, IgA, and IgM) also decreased during the study. The serum IgG level decreased significantly from 731.92 ± 180.54 mg/dl at baseline to 682.59 ± 189.98 and 684.37 ± 184.57 mg/dl on days 56 and 84, respectively (*p* < 0.05, Figure 4b); the level recovered to 690.88 ± 191.31 and 761.95 ± 252.36 mg/dl by days 112 and 168, respectively. The changes in serum IgA level were similar to those for serum IgG level (Figure 4c). On the other hand, serum IgM level decreased significantly from 69.87 ± 33.97 mg/dL at baseline to 64.21 ± 35.34 mg/dl on day 28 and remained significantly lower than baseline throughout the study (Figure 4d).

Univariate analysis was performed to identify the baseline characteristics affecting the treatment response, including clinical type of pemphigus, age, body weight, disease duration, corticosteroid dose, PDAI score, serum anti‐Dsg autoantibody levels, and presence or absence of adjuvant immunosuppressants; however, none of the aforementioned factors were associated with treatment response (Table S1). In addition, there were no differences in baseline characteristics between patients who did (*n* = 15) and did not (*n* = 5) achieve remission (Figure S1). ADA was detected in serum samples collected on day 168 in four patients (20%). Of these patients, three achieved CR with low levels of peripheral blood B cells and anti‐Dsg autoantibody levels throughout the study. In one patient, the serum rituximab level was below the detection limit on day 56, and the serum anti‐Dsg autoantibody titers gradually increased although peripheral blood B cells were not detected until the end of the study. Although the PDAI score decreased from 27 to 17 by day 168, the patient did not achieve remission.

### 3.3 Adverse events

A total of 70 adverse events (AEs) were reported in 17 of 20 patients (85.0%; Table 2), of which 66 (94.3%) were grade 1 or 2 (mild or moderate). Grade 3 AEs occurred in three patients (15%), including one patient (5%) with cataract, one patient (5%) with pneumonia, and one patient (5%) with osteonecrosis and bacterial arthritis. No grade ≥4 AEs (life‐threatening or fatal) were reported. Four serious AEs (SAEs) requiring hospitalization or considered otherwise medically significant occurred in two patients (pneumonia in one patient [5%] and heart failure, radial fracture, and osteonecrosis in one patient [5%]).

**TABLE 2:** Summary of adverse events

	Events	Number of events	Number of patients
AEs		70	17
Grade 1 or 2		66	14
Grade 3		4	3
Grade ≥4		0	0
Serious		**4**	**2**
Drug‐related AEs (ADRs)		9	5
Grade 1 or 2			
Cardiac disorders	Palpitations	2	1
General disorders and administration site conditions	Pyrexia	1	1
Immune system disorders	Hypogammaglobulinaemia	1	1
Injury, poisoning and procedural complications	Infusion‐related reaction	2	1
Investigations	Gamma‐glutamyltransferase increased	1	1
	Immunoglobulins decreased	1	1
Grade 3/Serious			
Infections and infestations	Pneumonia	1	1

Abbreviations: AE, adverse event; ADR, adverse drug reaction.

Among the 70 AEs, nine were possibly related to rituximab treatment (adverse drug reaction) and developed in five patients (25%). Eight of nine AEs were grade 1 or 2 and included palpitations (two events in one patient), pyrexia, hypogammaglobulinaemia, infusion‐related reaction (two events in one patient), elevated gamma‐glutamyl transferase level, and decreased immunoglobulin levels. All AEs resolved without treatment, except for one patient who required IgG supplementation. Of the four SAEs, pneumonia was the only event considered to have a causal relationship with rituximab. The patient with pneumonia was unable to continue the trial and rituximab administration on day 14 was canceled, although the event improved with conventional supportive care and antibiotics. Sixty‐one of 70 AEs were considered to be unrelated to rituximab but related to concomitant medications or coincidental events. Unrelated AEs observed in more than two were nasopharyngitis (10 events), toxic skin eruption, conjunctivitis, folliculitis, blood urea increased (three events each), hepatic function abnormality, dental caries, headache, and abdominal pain (two events each), all of which were grade 1 or 2 (mild or moderate) and resolved with appropriate treatments. No AEs associated with ADA were observed.

## 4 DISCUSSION

The present study was conducted in Japan to evaluate the efficacy and safety of rituximab in patients with refractory pemphigus who failed to achieve remission with conventional therapies. Overall, 75% of the 20 patients achieved remission with minimal therapy (55% CR and 20% PR). In addition, the PDAI score improved, serum anti‐Dsg autoantibody levels decreased, and corticosteroid dose was successfully reduced from baseline in all cases. Although 85% of patients experienced AEs, most were not serious and four SAEs were appropriately handled, indicating no significant risk from rituximab treatment in patients with pemphigus. Our results provide evidence for the use of rituximab for the treatment of pemphigus.

Although the present study had several limitations, including its open‐label, uncontrolled, single‐arm design and small number of included patients, our results are consistent with those of previously published prospective studies that have demonstrated the clinical efficacy of rituximab in patients with pemphigus.^21^, ^22^ The first prospective trial of rituximab in pemphigus was reported in 2007; of 21 patients with corticosteroid‐refractory disease, a single course of rituximab (375 mg/m^2^ weekly for four doses) showed a response rate of 86% (18 of 21 patients) after 3 months of the final rituximab dose with successful prednisone dose tapering.^23^ The Ritux 3 (First‐Line Rituximab Combined With Short‐Term Prednisone Versus Prednisone Alone for the Treatment of Pemphigus) trial published in 2017 compared rituximab plus short‐term prednisone and prednisone alone as the first‐line induction treatment of patients with newly diagnosed moderate to severe pemphigus.^18^ In all, 90 patients were randomized and 89% of patients in the rituximab plus short‐term prednisone group achieved CR off‐therapy compared with 34% in the prednisone alone group at 24 months. PEMPHIX (A Randomized, Double‐Blind, Double‐Dummy, Active‐Comparator, Multicenter Study to Evaluate the Efficacy and Safety of Rituximab Versus MMF in Patients With Pemphigus Vulgaris) was published in 2021 and compared rituximab with mycophenolate mofetil (MMF) for remission induction in patients with moderate to severe PV.^24^ In total, 138 patients were enrolled, and rituximab was superior to MMF, with 40% of rituximab‐treated patients achieving sustained CR without the use of corticosteroids for at least 16 consecutive weeks, compared with 10% in the MMF group, at 52 weeks. Rituximab treatment was associated with a greater reduction in glucocorticoid dose compared with MMF. The administration protocols of rituximab and corticosteroids, as well as the evaluation methods, have varied among previous studies, which makes it difficult to compare results. However, we found that 75% of patients achieved CR or PR on minimal therapy at 24 weeks, which is comparable to the findings of previous prospective studies.^25^

Despite the high incidence (85%) of AEs in the present study, no grade ≥4 SAEs were noted, and the AEs noted were not severe compared with previously reported AEs. The safety of rituximab needs careful consideration because of the report of two deaths caused by pyelonephritis and septicemia in the 2007 study.^23^ However, the Ritux 3 trial in 2017 did not report any deaths and demonstrated a significantly lower incidence of SAEs in the rituximab + prednisone group compared with the prednisone alone group.^18^ Similarly, the PEMPHIX trial in 2021 reported a similar incidence of AEs between the rituximab (85%) and MMF (88%) groups, with slightly higher incidences of SAEs and serious infections in the rituximab group (22% and 9%) compared with the MMF group (15% and 6%).^24^ There were no deaths in the rituximab group during the study, whereas one patient in the MMF group died of lung cancer. Based on recent reports, including the present study, rituximab appears to be safe for the treatment of pemphigus. In addition to infections, an important AE of rituximab therapy in patients with pemphigus is infusion‐related reactions, which had an incidence of 5% (one of 20 patients) in the present study and was not observed in a prior study conducted in Japan in 2019.^19^ On the other hand, infusion‐related reactions occurred in 22% of patients (15 of 67) in the PEMPHIX trial.^24^ The discrepancy between the results of the present and previous studies may be attributable to differences in the diagnostic criteria for infusion‐related reactions. However, a possible role of other factors, such as race, cannot be excluded and needs to be examined in future studies.

Similar to previous studies, rituximab treatment was effective for most patients with pemphigus in the present study. Because some patients did not achieve remission, it would be helpful to identify predictive factors for response to rituximab treatment. In the present study, there were no differences in the baseline characteristics between patients who did and did not achieve remission, suggesting that rituximab may be effective for all patients with pemphigus. The inadequate efficacy of rituximab treatment in some patients may be related to the presence of ADA.^26^, ^27^ In our study, one patient demonstrated reduced blood rituximab level after ADA was detected and serum anti‐Dsg autoantibody titers were increased, resulting in failure to achieve remission. In the PEMPHIX trial, ADA was detected in 31.7% of patients (20 of 63), with no differences in the efficacy and safety of rituximab between patients with and without ADA.^24^ In our study, ADA was detected in 20% of patients (four of 20) and no differences were observed in the rate of remission between patients with and without ADA.

Although there are still improvements to be made, such as countermeasures against recurrence and relapse, we will need to mature in the careful use of rituximab as the mainstay of future pemphigus treatment strategies.

## CONFLICT OF INTEREST

Naomi Mashino and Masahiro Abe are employees of Zenyaku Kogyo Co., Ltd. Masayuki Amagai and Jun Yamagami have received research funding, lecture fees, and consultancy fees from Zenyaku Kogyo Co. Ltd. Rituximab used in this trial was provided, free of charge, by Zenyaku Kogyo Co. Ltd. The company partially provided financial support for the trial although it was not involved in determining or interpretation of the results.

## Supporting information


Figure S1

Table S1
