# Supplementary figures and images for: Rituximab therapy for intractable pemphigus: A multicenter, open‐label, single‐arm, prospective study of 20 Japanese patients
Source: J Dermatol. 2022 Oct 4;50(2):175–82. doi: 10.1111/1346-8138.16597 (PMC10091989; doi:10.1111/1346-8138.16597)

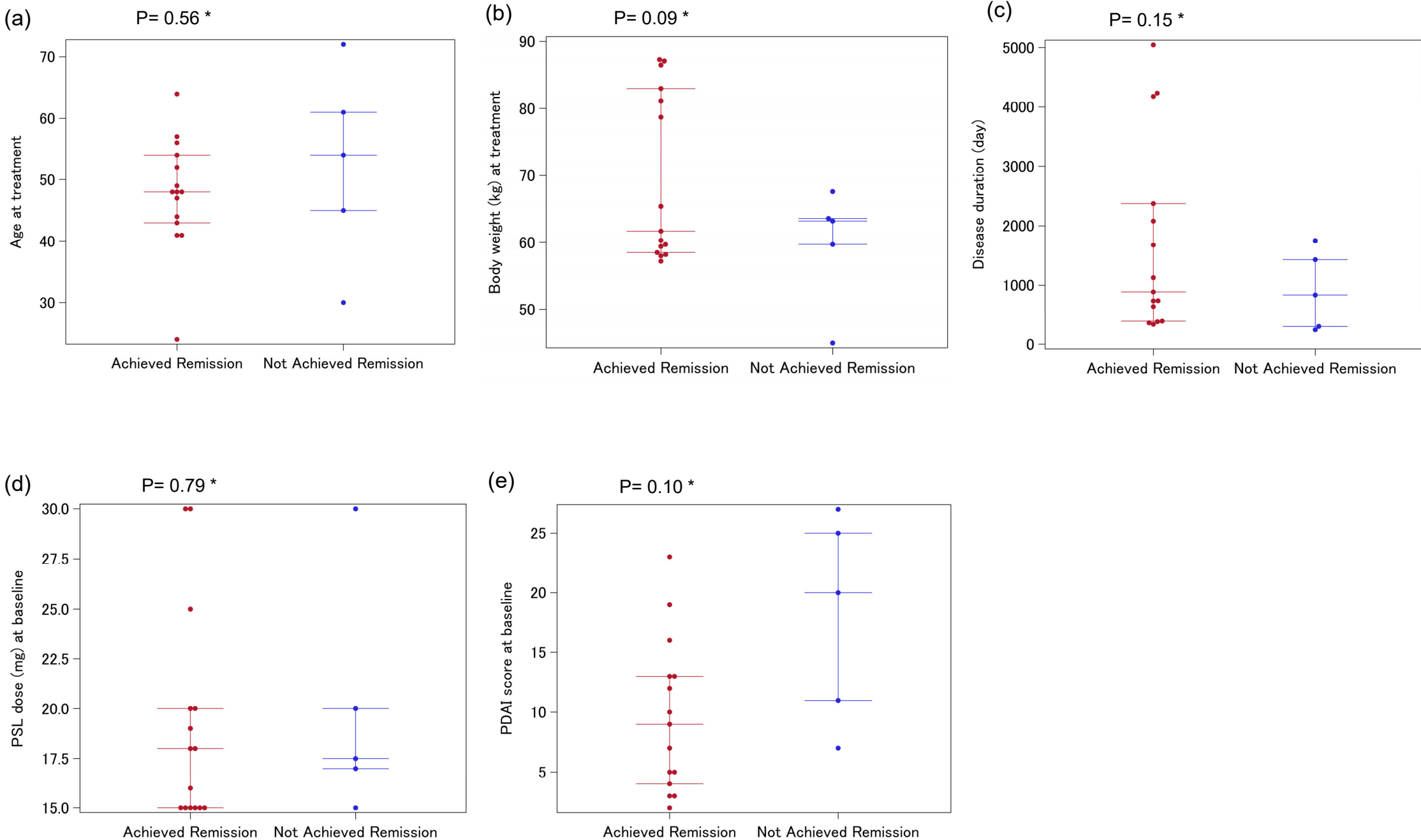

\*Welch's T-test

Supplementary Figure 1

Supplement: Supplementary file 1 — Figure S1 [file JDE-50-175-s002.pdf]
