# Supplementary material for: Rituximab therapy for intractable pemphigus: A multicenter, open‐label, single‐arm, prospective study of 20 Japanese patients
Source: J Dermatol. 2022 Oct 4;50(2):175–82. doi: 10.1111/1346-8138.16597 (PMC10091989; doi:10.1111/1346-8138.16597)
Supplement: Supplementary file 2 — Table S1 [file JDE-50-175-s001.pdf]

	remission rate %	P-value*
Overall response	75.0 (15/20)	-
CR	55.0 (11/20)	
PR	20.0 (4/20)	
Factors		
Type of pemphigus		
PV	72.7 (8/11)	
PF	75.0 (6/8)	1.000
Age at treatment (median 48 years old)		
< 48	75 (6/8)	
≥ 48	75 (9/12)	1.000
Body weight at treatment (median 62.45 kg)		
< 62.45 kg	80.0 (8/10)	
≥ 62.45 kg	70.0 (7/10)	1.000
Disease duration (median 860 days)		
< 860 days	70.0 (7/10)	
≥ 860 days	80.0 (8/10)	1.000
PSL dose at baseline (median 17.75 mg/day)		
< 17.75 mg/day	70.0 (7/10)	
≥ 17.75 mg/day	80.0 (8/10)	1.000
PDAI score at baseline		
≤ 8 (mild)	87.5 (7/8)	
≥ 9 (moderate to severe)	66.7 (8/12)	0.603
anti-Dsg1 antibody at baseline (median 276 U/mL)		
< 276 U/mL	88.9 (8/9)	
≥ 276 U/mL	66.7 (6/9)	0.576
anti-Dsg3 antibody at baseline (median 499 U/mL)		
< 499 U/mL	80.0 (4/5)	
≥ 499 U/mL	60.0 (3/5)	1.000
Concomitant immunosuppressants		
Yes	73.3 (11/15)	
No	80.0 (4/5)	1.000

* Fisher's exact test

Supplementary Table 1
